# Supplementary material for: Deletion of wfs1 Impairs Oligodendrocyte Precursor Cells Dorsal Distribution and Myelination Through the wfs1-hmgcs1 Axis in Zebrafish
Source: Biology (Basel). 2026 Aug 16;15(16):1402. doi: 10.3390/biology15161402 (PMC13510151; doi:10.3390/biology15161402)
Supplement: Supplementary file 1 [file biology-15-01402-s001.zip › File S1-Transcriptome sequencing data and differentially expressed genes (DEGs) in wfs1-- zebrafish at 5 dpf/2.KEGG/wfs1KOvsWT/UP/src/dre04621.html]

dre04621


- dre:566185

- Up regulated genes

566185(6.72123660882883)

- dre:566185

- Up regulated genes

566185(6.72123660882883)

- dre:566185

- Up regulated genes

566185(6.72123660882883)

- dre:566185

- Up regulated genes

566185(6.72123660882883)

- dre:373874

- Up regulated genes

110437957(1.43280591760355)

- dre:566185

- Up regulated genes

566185(6.72123660882883)

- dre:566185

- Up regulated genes

566185(6.72123660882883)

- dre:562552

- Up regulated genes

562552(1.50682290794657)

- dre:557239

- Up regulated genes

557239\_1(1.35743652020669)

- dre:436718

- Up regulated genes

436718(1.34229427553561)

- dre:393186

- Up regulated genes

393186\_1(3.79579966743607)

- dre:326974

- Up regulated genes

326974\_1(1.07837684837292)

- dre:566185

- Up regulated genes

566185(6.72123660882883)

- dre:566185

- Up regulated genes

566185(6.72123660882883)

- dre:566185

- Up regulated genes

566185(6.72123660882883)

- dre:566185

- Up regulated genes

566185(6.72123660882883)

- dre:373874

- Up regulated genes

110437957(1.43280591760355)

- dre:561021

- Up regulated genes

561021(2.14284154145004)

- dre:566185

- Up regulated genes

566185(6.72123660882883)

- dre:796505

- Up regulated genes

110438502(2.03997015305697)

- dre:569298

- Up regulated genes

569298(1.32871616109562)

Close
